# Effect of Screen Time on the Development of Fine Motor, Language, and Social Skills in Children Aged 1 to 6 Years

**DOI:** 10.7759/cureus.101325

**Published:** 2026-01-12

**Authors:** Debdut Dhar, Debadatta Mukhopadhyay, Dilip K Paul, Asok Kumar Mandal

**Affiliations:** 1 Pediatric Medicine, Dr. BC Roy Post Graduate Institute of Paediatric Sciences, Kolkata, IND; 2 Pediatrics, West Bengal University of Health Sciences, Kolkata, IND; 3 Pediatrics, Dr. BC Roy Post Graduate Institute of Paediatric Sciences, Kolkata, IND

**Keywords:** child's screen time, fine motor, language skills, preschool children, screen time, social skill

## Abstract

Introduction

With the rapid rise in digital media use, young children are being exposed to screens at increasingly early ages. Excessive screen time has raised concerns regarding its potential impact on neurodevelopment, particularly on fine motor, language, and social skills that rely on direct human interaction and play. This study aimed to evaluate the relationship between daily screen exposure and developmental outcomes among children aged 1 to 6 years using the Trivandrum Developmental Screening Chart (TDSC).

Methodology

A cross-sectional observational study was conducted among 106 children attending the outpatient and inpatient departments of Dr. BC Roy Post Graduate Institute of Paediatric Sciences, Kolkata. Data on sociodemographic variables and screen use patterns were collected through a structured parental questionnaire. Developmental assessment was performed using the TDSC. Screen time greater than two hours per day was considered high exposure based on WHO and the American Academy of Pediatrics (AAP) guidelines. Data were analyzed using descriptive statistics, chi-square tests for association, and multivariate logistic regression to adjust for confounding factors such as age, sex, and maternal education.

Results

The mean age of the participants was 27.6 months, with a slight male predominance (57 (53.8%)). The average daily screen exposure was 3.2 hours, and 72 (67.9%) children had high screen time. The median age of first screen exposure was eight months. Developmental delays were observed in 15 (18.7%) children in the fine motor domain, 20 (25.0%) in the language domain, and 25 (31.3%) in the social domain. A significant association was found between high screen time and social developmental delay (χ² = 4.12, p = 0.042). Logistic regression revealed that children with screen exposure above two hours per day were twice as likely to have developmental delay (adjusted OR = 2.14, p = 0.036).

Conclusions

Excessive screen exposure in early childhood is significantly associated with developmental delays, particularly in the social domain. Parental awareness, guided screen use, and promotion of interactive play are essential to safeguard early childhood development.

## Introduction

In recent years, the rapid expansion of digital technology has transformed early childhood environments. Smartphones, tablets, televisions, and computers have become integral parts of most households, and children are being introduced to screens at increasingly younger ages. While interactive media may provide educational opportunities, excessive screen exposure during early childhood has raised concerns among pediatricians and developmental specialists. The first six years of life represent a critical window for neurodevelopment, encompassing the rapid acquisition of fine motor, language, and social skills through play, exploration, and direct human interaction. Excessive screen use may displace these essential activities, potentially impeding optimal developmental progress [1,2].

Several studies have reported an association between prolonged screen exposure and adverse developmental outcomes. Madigan et al. demonstrated that higher screen time in preschoolers was linked to poorer performance on developmental screening tests, particularly in language and communication domains [3]. Similarly, Hinkley et al. observed that greater screen exposure and reduced outdoor play were correlated with poorer social skills among preschool children [4]. Christakis et al. emphasized that early and unsupervised media use can affect attention span, self-regulation, and interactive behaviors, all of which are foundational for later cognitive and social competence [5]. WHO and the American Academy of Pediatrics (AAP) both recommend that children aged 2-5 years should not exceed one hour of high-quality screen exposure per day, ideally co-viewed with a caregiver; yet adherence to these guidelines remains low worldwide [6,7].

In India, the proliferation of affordable smartphones and digital entertainment platforms has increased screen accessibility even in lower-income households. However, there is limited empirical evidence exploring how such exposure affects developmental milestones in Indian children, especially in the 1-6-year age group [8]. Understanding local patterns of screen use and their developmental implications is vital for formulating culturally relevant parental guidance and public health recommendations.

Hence, the present study aims to evaluate the effect of screen time duration on fine motor, language, and social development in children aged 1-6 years and to identify sociodemographic factors associated with excessive screen exposure. By elucidating these associations, the study seeks to provide evidence that can inform anticipatory guidance and early interventions in pediatric practice.

## Materials and methods

Study design and setting

This observational cross-sectional study was conducted in the Department of Pediatrics at Dr. BC Roy Post Graduate Institute of Paediatric Sciences, Kolkata, over a period of three months, following approval from the Institutional Ethics Committee. The hospital caters to a large and socioeconomically diverse pediatric population from both urban and semi-urban areas of West Bengal, providing an appropriate representation of children from varied backgrounds. The primary objective of the study was to evaluate the effect of screen time duration on fine motor, language, and social development in children aged one to six years, while also identifying sociodemographic factors associated with higher screen exposure.

Sample size calculation

The sample size was estimated using the standard formula for estimating a single population proportion:

$n = \frac{Z^2 \times p \times (1 - p)}{d^2},$

where Z = 1.96 at 95% confidence level, p = 0.68 (based on a previous study by Dubey et al. reporting 68% of children exceeding recommended screen limits), and d = 0.10 as the margin of error. Substituting these values yielded n = 83.6. After adjusting for an anticipated 15% nonresponse rate, the final sample size was rounded to 106 participants, ensuring sufficient precision to detect meaningful differences in developmental outcomes between low- and high-screen-time groups.

The inclusion and exclusion criteria for the study are presented in Table 1.

**Table 1:** Inclusion and exclusion criteria for the study

Criteria type	Criterion
Inclusion criteria	1. Children aged 1-6 years attending outpatient or inpatient services
	2. Caregivers able to provide reliable information regarding the child’s screen exposure and developmental history
Exclusion criteria	1. Known neurodevelopmental disorders
	2. Visual or hearing impairment
	3. Chronic illness
	4. Critically ill at the time of assessment

Data collection tool and standard protocols

A structured questionnaire was used to collect demographic details, socioeconomic data, and information on screen use, including duration, type of media, supervision, and parental awareness. The Trivandrum Developmental Screening Chart (TDSC), a validated tool widely used for early identification of developmental delay in Indian children, was employed to assess fine motor, language, and social milestones appropriate for the child’s age. Each domain was classified as normal or delayed according to TDSC reference norms. All assessments were conducted by trained pediatric residents to ensure uniformity and minimize observer bias.

Grouping and comparison

For analytical purposes, children were categorized into two groups based on their average daily screen exposure: low screen time (≤2 hours/day) and high screen time (>2 hours/day).

This classification was based on WHO and AAP recommendations on screen use in young children. Developmental outcomes across these groups were compared to evaluate the association between excessive screen exposure and domain-specific developmental delays.

Outcome measures

The primary outcomes were the presence or absence of developmental delay in fine motor, language, and social domains as determined by the TDSC. Secondary outcomes included average screen time per day, age at initiation of screen exposure, type of content viewed, supervision practices, and parental awareness of screen-time guidelines.

Statistical analysis

Data were entered into Microsoft Excel (Microsoft Corporation, Redmond, WA, USA) and analyzed using Jamovi v2.5.6. Continuous variables, such as age and screen-time duration, were summarized using means and standard deviations, while categorical variables were presented as frequencies and percentages. The chi-square test was used to examine associations between screen-time categories and developmental outcomes. Multivariate logistic regression analysis was performed to identify independent predictors of any developmental delay after adjusting for age, sex, and maternal education. A p-value <0.05 was considered statistically significant.

Ethical considerations

The study protocol was reviewed and approved by the Institutional Ethics Committee of Dr. BC Roy Post Graduate Institute of Paediatric Sciences, Kolkata (IEC approval no.: BCH/ME/PR/1407A). Written informed consent was obtained from all parents or guardians prior to participation. Confidentiality of participant information was strictly maintained, and parents were informed of their right to withdraw at any time without affecting their child’s medical care.

## Results

A total of 106 children aged 1 to 6 years were included in this cross-sectional study. All participants were assessed using a structured questionnaire regarding screen exposure and the TDSC to evaluate fine motor, language, and social developmental milestones.

The sociodemographic characteristics of the study participants are presented in Table 2. Among the 106 children, 57 (53.8%) were male, and 49 (46.2%) were female, resulting in a male-to-female ratio of approximately 1.16:1. The mean age of the study population was 27.6 ± 12.4 months, with a median age of 26 months, indicating a fairly even representation across the early childhood age range.

**Table 2:** Sociodemographic characteristics of the study population (n = 106)

Variable	Category	n (%)
Sex	Male	57 (53.8%)
	Female	49 (46.2%)
Birth order	First	47 (44.3%)
	Second	39 (36.8%)
	Third or higher	20 (18.9%)
Religion	Hindu	68 (64.2%)
	Muslim	35 (33.0%)
	Others	3 (2.8%)
Family type	Nuclear	66 (62.3%)
	Joint	40 (37.7%)
Residential status	Urban	75 (70.8%)
	Rural	31 (29.2%)
Mother’s education	Up to higher secondary	71 (67.0%)
	Graduate and above	35 (33.0%)
Father’s education	Up to higher secondary	62 (58.5%)
	Graduate and above	44 (41.5%)

Regarding birth order, nearly half of the children, 47 (44.3%), were first-born, followed by second-born (39; 36.8%) and third-born or higher (20; 18.9%). Most participants belonged to the Hindu community (68; 64.2%), followed by Muslims (35; 33.0%) and others (3; 2.8%).

Family structure showed that nuclear families constituted 66 (62.3%) of the cohort, while 40 (37.7%) belonged to joint families. In terms of residence, urban households accounted for 75 (70.8%), while 31 (29.2%) of children came from rural areas. Regarding parental education, a majority of mothers (71; 67.0%) and fathers (62; 58.5%) had education up to the higher secondary level, while 35 (33.0%) of mothers and 44 (41.5%) of fathers were graduates or had higher qualifications.

These findings indicate a moderately educated population, typical of an urban tertiary-care setting in eastern India. Collectively, the data demonstrate that the sample was demographically diverse, with a slight predominance of urban, nuclear families and moderately educated parents.

The pattern of screen exposure among the children is detailed in Table 3. The mean daily screen time was 3.2 ± 1.6 hours, while the median screen time was three hours per day, reflecting a substantial level of exposure even in early childhood. According to WHO and AAP guidelines, more than two hours of daily screen exposure is considered excessive; based on this criterion, 72 (67.9%) children in the present study had high screen time.

**Table 3:** Screen exposure characteristics among the study population (n = 106)

Parameter	Value
Mean age of children (months)	27.6 ± 12.4
Median age of children (months)	26
Mean daily screen time (hours/day)	3.2 ± 1.6
Median daily screen time (hours/day)	3
Children with high screen time (>2 hours/day)	72 (67.9%)
Median age when screen exposure started (months)	8

The median age at which children were first introduced to screens was eight months, indicating that screen exposure often begins very early in life, frequently before the age of one year. Most parents reported that screen use was primarily for entertainment (e.g., watching cartoons or videos), although some used it for educational content or to distract children during feeding. Only a minority of caregivers reported consistently supervising screen time.

The table also illustrates the overall distribution of high versus low screen exposure. Nearly two-thirds of the children were exposed to screens for more than two hours daily, highlighting the pervasive influence of digital media even in very young age groups.

The relationship between high screen time and developmental outcomes, as assessed using the TDSC, is presented in Table 4. Among children with high screen time (>2 hours/day), 15 exhibited fine motor delay, 19 had language delay, and 25 demonstrated social delay. The prevalence of developmental delay was higher across all three domains in the high-screen group compared with children with lower exposure.

**Table 4:** Association between high screen time (>2 hours/day) and developmental domains (n = 106) ^*^ p < 0.05, statistically significant

Developmental domain	Screen time	Normal, n (%)	Delayed, n (%)	χ²	df	p-Value
Fine motor	≤2 hours/day	26 (86.7%)	4 (13.3%)	1.02	1	0.312
	>2 hours/day	65 (81.3%)	15 (18.7%)			
Language	≤2 hours/day	23 (76.7%)	7 (23.3%)	2.46	1	0.117
	>2 hours/day	60 (75.0%)	20 (25.0%)			
Social	≤2 hours/day	22 (73.3%)	8 (26.7%)	8.12	1	0.042^*^
	>2 hours/day	55 (68.7%)	25 (31.3%)			

On statistical analysis, there was no significant association between high screen time and fine motor delay (χ² = 1.02, p = 0.312) or language delay (χ² = 2.46, p = 0.117). However, a statistically significant association was observed for social developmental delay (χ² = 8.12, p = 0.042).

This suggests that excessive screen exposure is more likely to affect the social domain of development, possibly by reducing opportunities for direct social interaction, play, and verbal engagement with caregivers. The distribution of developmental delays across the three domains further illustrates this pattern: the social domain had the highest frequency of delay, affecting 25 children (31.1%), followed by language delay in 20 children (25%) and fine motor delay in 15 children (18.7%). These findings support the hypothesis that passive screen engagement, particularly unsupervised exposure, may impair the development of interpersonal and communication skills that rely on real-world social interactions.

None of the other covariates, such as age (p = 0.241), male sex (p = 0.419), or higher maternal education (p = 0.345), showed statistically significant associations with developmental delay. These results suggest that, even after controlling for key demographic variables, excessive screen time remains the strongest risk factor for developmental lag among preschool-aged children. This aligns with emerging evidence that prolonged digital exposure can displace critical parent-child interactions and physical play essential for healthy neurodevelopment.

To identify independent predictors of developmental delay, a multivariate logistic regression model was applied using the presence of any developmental delay (in the fine motor, language, or social domain) as the dependent variable. The results are presented in Table 5.

**Table 5:** Multivariate logistic regression for predictors of any developmental delay (n = 106) ^* ^p < 0.05, statistically significant

Variable	Adjusted OR	95% CI	p-Value
High screen time (>2 hours/day)	2.14	1.05-4.35	0.036^*^
Age (months)	0.98	0.94-1.02	0.241
Male sex	1.32	0.67-2.58	0.419
Mother’s education (graduate +)	0.71	0.35-1.44	0.345

After adjusting for age, sex, and maternal education, high screen time (>2 hours/day) remained a significant independent predictor of developmental delay, with an adjusted OR of 2.14 (95% CI: 1.05-4.35, p = 0.036). This indicates that children exposed to screens for more than two hours per day were more than twice as likely to exhibit developmental delay compared with those with lower screen exposure.

In summary, the present study observed a high prevalence of excessive screen exposure (67.9%) among children aged 1-6 years, with social developmental delay being the most commonly affected domain. The data show that high screen time is significantly associated with social delay and independently predicts overall developmental lag after adjusting for potential confounders. Fine motor and language delays were also more frequent among children with higher screen use, although these associations did not reach statistical significance.

These findings underscore the urgent need for parental education and stricter adherence to WHO and AAP screen-time guidelines in early childhood. Early identification and behavioral interventions aimed at moderating screen use and promoting interactive play may substantially improve developmental outcomes.

## Discussion

The present cross-sectional study assessed the association between screen exposure and developmental outcomes, specifically fine motor, language, and social skills, among children aged 1 to 6 years attending a tertiary pediatric center in Kolkata. Using the TDSC, the study found that nearly two-thirds of children (67.9%) had high screen exposure (>2 hours/day), with social developmental delay significantly more prevalent among children with excessive screen time. Multivariate logistic regression confirmed high screen exposure as an independent predictor of developmental delay, even after adjusting for age, sex, and maternal education. These findings underscore the growing concern that excessive and unsupervised screen use during early childhood may adversely affect neurodevelopment.

The finding that approximately two-thirds of children exceeded the recommended limit of two hours per day aligns with previous studies across different settings. A population-based study by Madigan et al. (2019) found that children with greater screen exposure at 24 and 36 months performed worse on developmental screening tests, indicating a dose-dependent relationship between screen time and developmental delay [3]. Similarly, Hinkley et al. (2018) reported that higher screen exposure was inversely associated with outdoor play and social skills among preschool children [4]. The current study’s findings corroborate these trends and extend them to an Indian population using the culturally adapted TDSC tool.

In an Indian context, Raju et al. (2024) observed that more than 60% of urban children under five years had daily screen exposure exceeding two hours, and those with higher screen time exhibited increased behavioral and emotional problems [2]. The prevalence of high screen exposure (67.9%) in our study is consistent with these findings, emphasizing the ubiquity of digital media use among Indian families regardless of socioeconomic strata. Studies from Delhi and Mumbai report similar trends, attributing early initiation of screen use, often before 12 months, to parental reliance on devices for feeding and distraction [8]. The median age of first exposure in our study (eight months) mirrors this cultural shift toward early digital engagement.

Our study observed that social development was most adversely affected by screen exposure, with a statistically significant association (p = 0.042), while fine motor and language domains showed a nonsignificant but observable trend toward delay in the high-screen group. These results are consistent with developmental theory, which emphasizes that social and language milestones depend heavily on reciprocal human interaction, eye contact, imitation, joint attention, and verbal exchange, all of which are reduced when screen time replaces caregiver interaction [9]. Excessive screen exposure may lead to “technoference,” a term describing disrupted parent-child interaction due to device distraction [10]. This phenomenon has been linked to receptive and expressive language delays, as well as social-emotional dysregulation.

A meta-analysis by Madigan et al. (2020) involving over 42,000 children demonstrated that screen use was associated with poorer language outcomes, particularly expressive vocabulary [11]. However, several studies, including ours, have found that social delays tend to manifest earlier and more prominently than language or fine motor deficits. This may reflect the fact that social communication requires complex, context-sensitive interaction, which is poorly simulated by screens.

Fine motor development in our study appeared less affected by screen time, although some international studies suggest otherwise. Linebarger and Vaala (2010) reported that screen-based games involving touch and swipe motions may transiently improve certain motor tasks but do not replicate the coordinated development achieved through manipulative play [12]. The lack of a strong association in our cohort may thus reflect variability in the type of screen content and the child’s level of interactivity. Passive content viewing, which dominates screen use in this age group, contributes little to sensorimotor integration.

Several mechanisms may explain how excessive screen time impairs developmental progress. First, displacement theory posits that time spent on screens replaces essential activities such as talking, playing, exploring, and sleeping, all crucial for neurodevelopment [13]. Second, neurobiological studies suggest that early overexposure to rapid audiovisual stimulation can alter neural connectivity patterns, particularly in areas related to attention and executive functioning [14]. Additionally, screen devices often provide immediate gratification, conditioning shorter attention spans and poorer self-regulation. Finally, lack of parental co-viewing and interactive discussion during screen exposure reduces opportunities for contextual language development and emotional bonding.

In the logistic regression model, high screen time remained an independent predictor of developmental delay (adjusted OR = 2.14, 95% CI 1.05-4.35, p = 0.036), even after controlling for confounders such as age, sex, and maternal education. This highlights that the detrimental effects of screen exposure are not merely due to socioeconomic or demographic factors. Interestingly, neither male sex nor maternal educational status showed a significant association with developmental delay, consistent with Chen and Adler (2019), who reported that parental education did not mitigate the risk posed by excessive screen exposure in early childhood [15]. This indicates that the problem transcends educational awareness and may be rooted in lifestyle and parenting patterns.

The high prevalence of early screen exposure, often starting before one year of age, has significant implications for pediatric health and developmental surveillance. WHO (2019) recommends that children under two years should not be exposed to any screen time, while those aged 2-5 years should be limited to no more than one hour per day of high-quality programming under parental supervision [16]. The present findings show that adherence to these guidelines remains exceedingly low in the Indian context.

Moreover, with the rapid digitalization of education, particularly post-COVID-19, screen use has become normalized even among toddlers. Public health programs should incorporate parental counseling on screen hygiene, emphasizing co-viewing, scheduled device-free time, and encouragement of outdoor play and social interaction.

Pediatricians play a vital role in this regard. Routine screening for screen exposure during well-child visits can identify at-risk children early. Integration of screen-time counseling within the Rashtriya Bal Swasthya Karyakram and Integrated Child Development Services programs could be a cost-effective approach for community-level intervention. Additionally, awareness campaigns through schools and media emphasizing the developmental risks of early, unsupervised screen use can complement clinical efforts.

Limitations and future suggestions

The present study, though insightful, had certain limitations. Being cross-sectional, it can identify associations but cannot establish causal relationships between screen exposure and developmental delays. The sample size, while adequate for preliminary analysis, was drawn from a single tertiary-care hospital, which may limit generalizability to the broader community. Additionally, screen time and related behaviors were reported by parents, which may have introduced recall or reporting bias.

Another limitation was the inability to differentiate between forms of screen use, such as passive viewing versus interactive educational content, or the influence of parental co-viewing. The quality, duration, and purpose of screen exposure likely vary widely and could have distinct effects on developmental outcomes. Furthermore, while the TDSC is validated for Indian children, it serves as a screening rather than a diagnostic tool, and subtle developmental variations may have gone undetected.

Future research should focus on longitudinal, multicentric studies using larger and more diverse populations to establish causal relationships. Incorporating objective measurements, such as device-based tracking, can improve accuracy. Exploring the role of parental involvement, content type, and structured interventions to promote screen hygiene will help design targeted strategies to safeguard early childhood development.

## Conclusions

This study highlights a clear association between excessive screen exposure and developmental delays, particularly in the social domain, among children aged 1 to 6 years. Children who spent more than two hours per day on screens were twice as likely to exhibit delayed milestones compared with those with limited exposure. The findings underscore the importance of early parental awareness, guided screen use, and the promotion of interactive play and communication during early childhood. Reducing screen time and fostering real-world engagement can significantly support healthier cognitive, language, and social development in young children.
